# Cross-sectional seasonal prevalence and relative risk of ectoparasitic infestations of rodents in North Sinai, Egypt

**DOI:** 10.14202/vetworld.2021.2996-3006

**Published:** 2021-11-27

**Authors:** Doaa S. Farid, Nahla H. Sallam, Ahmed M. Salah Eldein, Essam S. Soliman

**Affiliations:** 1 Department of Environmental Protection, Faculty of Environmental Agricultural Sciences, Arish University, Arish 45516, Egypt; 2 Department of Parasitology, Faculty of Veterinary Medicine, Suez Canal University, Ismailia 41522, Egypt; 3 Department of Wildlife and Zoo, Faculty of Veterinary Medicine, Suez Canal University, Ismailia 41522, Egypt; 4 Animal, Poultry, and Environmental Hygiene Division, Department of Animal Hygiene, Zoonosis, and Animal Behavior, Faculty of Veterinary Medicine, Suez Canal University, Ismailia 41522, Egypt

**Keywords:** ectoparasites, North Sinai, period prevalence, relative risk, rodents

## Abstract

**Background and Aim:**

Rodents are ubiquitous animals that host ectoparasites and transmit zoonotic diseases. We conducted a cross-sectional study on the seasonal variation, period prevalence (Pp), and relative risk of ectoparasitic infestations in rodents collected in North Sinai, Egypt, from September 2019 to August 2020.

**Materials and Methods:**

We captured 380 rodents during the study period. Rodents were euthanized to perform species identification, and 2930 external parasites were collected and identified using light microscopic examination with systemic keys depending on morphological characters.

**Results:**

*Rattus norvegicus* (brown rat), *Rattus rattus frugivorus* (white-bellied rat), *Rattus rattus alexandrines* (gray-bellied rat), and *Mus musculus* *domesticus* (house mouse) were captured at the highest frequencies during summer (n=186), followed by spring (n=84), fall (n=71), and winter (n=39), with a higher proportion of males captured in all seasons. Analysis of the infestation Pp revealed highly significant increases (p<0.01) in ectoparasites during the winter. Temperature, humidity, and dew point were significantly (p<0.01) correlated with the numbers of captured and infested rodents. Parasitological examinations showed the higher risks of flea (*Echidnophaga gallinacea*, *Xenopsylla cheopis*, and *Leptopsylla segnis*) and lice (*Hoplopleura hirsuta*, *Hoplopleura ocanthopus*, *Hoplopleura oenomydis*, and *Polyplax spinulosa*) infestations during winter and mite (*Laelaps nuttalli*, *Dermanyssus gallinae*, *Ornithonyssus bacoti*, and *Myobia musculi*) infestations during summer.

**Conclusion:**

We conclude that ectoparasitic infestation prevalence and risk varies with predominating macroclimatic conditions. Strict preventive and biosecurity measures should be applied to combat rodent-related problems.

## Introduction

Rodents are widespread nocturnal species, representing approximately 40% of all mammals [1]. Rodents are nearly ubiquitous and well-adapted to terrestrial areas from tundras to deserts, playing important ecological roles through their burrowing activities [2]. Rodents contribute to billions of dollars in property and food resource losses annually and negatively impact human and animal health [3]. Rodents are carriers or reservoirs for various viruses, bacteria, rickettsia, and helminths, responsible for transmitting many zoonotic and infectious diseases [4]. These diseases can be transmitted through direct exposure to rodent secretions and excretions or indirect exposure pathways through ectoparasites [5]. Rats are commensal, meaning they live in close proximity and “share tables” with humans; they are known for their adaptability to residential areas and hosting a wide range of ectoparasites [6,7].

Ectoparasites represent a wide variety of highly adaptive species-specific or wide range infective organisms that temporally or permanently habituate the body surfaces of animals [8,9]. Ectoparasites are vectors or reservoirs that transmit pathogenic agents (bacteria, viruses, protozoa, and helminths) to humans [10,11] and domestic animals [12], some of which present a zoonotic nature [13]. Ectoparasite populations are dependent on host-specific factors, such as population capacity, range, age, sex, behavior, and skin covering [14,15], environmental factors, such as temperature, relative humidity, dew point, geographical location, and seasonal variation [16], as well as the nutritional, developmental, and maturation requirements of the parasite [17].

Rodent ectoparasites are mainly classified as Acarina (ticks), Siphonaptera (fleas), Mesostigmata (mites), and Phthiraptera (lice). Fleas can provide a biological vector for microbial agents, such as plague-causing *Yersinia pestis*, Lyme- and relapsing fever-causing *Borrelia*, Salmonellosis-causing *Salmonella*, Tularemia-causing *Francisella tularensis*, trypanosomiasis-causing *Trypanosoma*, and leishmaniasis-causing *Leishmania* [18]. Mites are known to parasitize rodents (wild and commensal) and humans and transmit *Wuchareria bancrofti*, which causes filariasis or elephantiasis [19]. Lice also transmit plague, tularemia, and *Rickettsia typhi* [20].

Rodent ecology and distribution are important factors affecting infectious and zoonotic disease dynamics, risk of transmission, and development in rodents, animals, and humans [21,22]. The distribution and ecology of rodents and rodent ectoparasites that contribute to the high-risk transmission of infectious and zoonotic diseases in North Sinai, Egypt remains mysterious.

The current cross-sectional study investigated the ecological distribution of rodents to identify the species colonizing the study area in North Sinai governorate, Egypt. In addition, we conducted period prevalence (Pp) and risk assessments of ectoparasitic infestations in rodents collected from September 2019 to August 2020, considering seasonal variation.

## Materials and Methods

### Ethical approval

The Scientific Research Ethics Committee for the animal, poultry, and lab animal research, Faculty of Veterinary Medicine, Suez Canal University, Egypt, approved the materials, protocol, and study design (approval number 2021014).

### Study period and area

**Figure-1:**
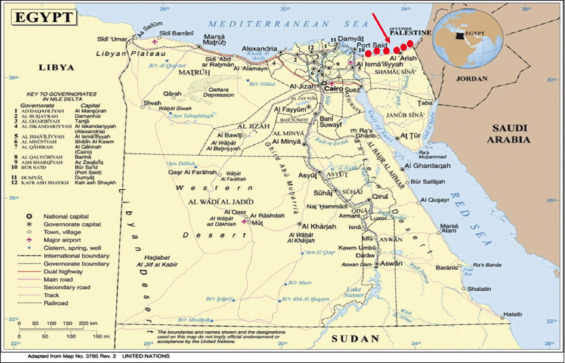
The geographical location of the total study area in North Sinai Governorate – Egypt [Source: General map of Egypt, March 2012 | UN Cartographic Section]. Red circles are located on the six geographical locations of the sampling areas.

The study was carried out from September 2019 to August 2020. The study was conducted in North Sinai, Egypt. The study area was located in the northeast (33.6176° E, 30.2824° N), bound to the north by the Mediterranean Sea with great dimensions up to 220 km (130 miles) from east to west. The study area was 27.574 km^2^, representing approximately 2.7% of Egypt’s area (Figure-1). North Sinai governorate is characterized by a unique Mediterranian climate, consisting of desert and semi-desert regions. Average temperatures and relative humidity vary from 10°C and 18% in the winter and 24°C and 96% in the summer. The northward region receives more rain, which lasts for 3.8 months (November 13-March 7) and accumulates an average of 20.3 mm total precipitation.

During the study period, four seasons of 3 months each were involved in the study. The duration of each season was defined concerning the general and predominating macroclimatic conditions as follows: Fall from September to the end of November, winter from December to the end of February, spring from March to the end of May, and summer season from June to the end of August.

### Study design

A cross-sectional retrospective study was designed to investigate the seasonal variation and geographical distribution of rodent species and their ectoparasites.

**Figure-2:**
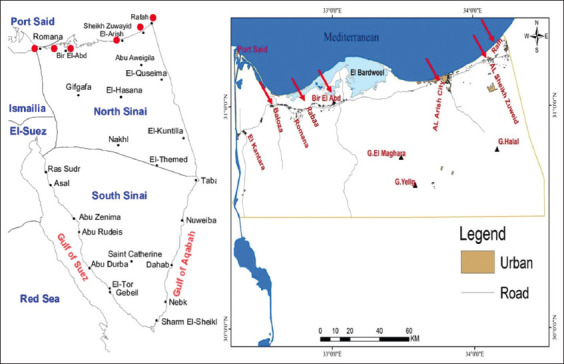
The geographical locations of the six sampling areas on the North Sinai governorate map – Egypt [Source: https://www.cambridge.org/core/journals/journal-of-biosocial-science/article/abs/prevalence-of-consanguineous-marriages-in-south-sinai-egypt/339AED57DFB936AF4838E81E3314C277, Cambridge University Press]. Red circles and arrows are located and pointed respectively on the six geographical locations of the sampling areas.

During the study period, a total of 300 wire traps were purchased in a monthly pattern and thoroughly cleaned using hot water and quaternary ammonium compound. Traps were baited with a variety of fresh foods, including dried fish, tomato, cheese, bread, and slices of cucumber. The trap baits were alternated from time to time to maximize the possibility of catching rodents and overcome the first suspicious impressions from the rodents. Wire traps were distributed in different geographical locations across the North Sinai governorate, such as Baloza, Rabaa, Bir el*-`*Abd, El-Arish, Sheikh Zuweid, and Rafah (Figure-2). The traps were positioned near residential buildings and poultry, sheep, and goat farms. Traps were distributed before sunset and recollected just before sunrise.

### Sampling

A total of 380 rodents were captured during the four successive seasons of the study period. All trapped rodents were enclosed in specially designed plastic bags before euthanasia to ensure the collection of the ectoparasites harbored on their bodies. The rodents were transported to laboratories for identification and external parasite collection.

### Rodent laboratory management and identification

In the laboratory, rodents were humanely euthanized using diethyl-ether to perform identification and ectoparasite sampling. Rodents were weighed using a digital scale. The sex and morphological characteristics were recorded, including the head, body, and tail length (mm) for rodent species identification according to Herbreteau *et al*. [23] and Rahdar and Vazirianzadeh [24]. After the collected rodent species were identified, the frequencies of each species were related to the total number of captured rodents, and seasonal variations during the study period were assessed as recommended by Thrusfield [25].

### Ectoparasite collection and identification

Ectoparasites were collected by brushing over the head, neck, trunk, tail, ears, around the eyes, and other parts of each rodent’s body using a fine brush. Visible ectoparasites, including those in the transportation bags, were quickly picked up using a toothbrush. Hair particles and ectoparasites were dropped onto a deep white dish or a white sheet of paper. The ectoparasites were counted, grouped by morphology, and fixed in 70% alcohol in sterilized screw-capped clear bottles marked with the date, area of collection, species, and sex of the rodents. Separate bottles were used for each animal host.

The preparation of ectoparasites for light microscopical examination was carried into about Farid *et al*. [26]. The screw-capped ectoparasite samples were placed into sodium hydroxide 10%, washed using distilled water several times, and dehydrated in serial dilution of alcohol constituting 25%, 50%, 75%, and 100% ethyl alcohol in concentrations. The ectoparasite samples were displaced into Xylene, mounted onto the glass slides with Canada balsam for fleas and lice and Berlese medium for mite, and covered by coverslips. The prepared slides were left for dryness in a hot oven at 40-50°C for 24 h and kept for examination under a light microscope (Barska^®^ AY13180 Binocular Stereo Microscope, B & H, NY, USA, 10× magnification).

The collected fleas and lice were identified after the available systematic keys of Hendrix [27] and Soulsby [28] and mite after Honey *et al*. [29] using different keys such as ctenidia, mesopleural rod, and occipital bristle for fleas; dorsal shield and setae for mite, and parategal plate for lice.

### Ecological measurement

Ecological macroclimatic conditions, including temperature, relative humidity, and air quality, were recorded regularly in a synchronized pattern with the rodent capture times using digital thermometers (ThermoPro^®^ TP50 Digital LCD Thermometer Hygrometer Temperature Humidity Meter, ThermoPro, GA, USA) and thermo-hygrometers (Digital Thermometer Hygrometer Indoor Outdoor Temperature Meter Humidity Monitor with LCD Alarm Clock, 3M Probe Cord, Kanbkam, UAE). The dew points were calculated using the temperature and relative humidity values following Lawrence [30]. The macroclimatic conditions were recorded during the four successive seasons of the study to detect relationships between prevailing weather conditions and parasitic infestations.

### Epidemiological measures

We calculated Pp and relative risk (RR) as described by Thrusfield and Christley [31]. We calculated the Pp for the total infestation, fleas, lice, and mites, according to rodent species, rodent sex, and season as follows:

Period prevalence (Pp) rate = (α/µ)×100

Where α is the number of (total/specific) infested rodents during a specific period and µ is the number of susceptible rodents in the population (species/sex-specific) during the same period.

RR, that is, the risk of contracting a specific infestation (flea, lice, and mite) during a specific season in the exposed rodent population, was calculated as follows:

RR = [((a/(a+b))/((c/(c+d))]

Where ((a/(a+b)) is the risk that the exposed develop a disease and ((c/(c+d)) is the risk that the non-exposed develop a disease.

### Statistical analysis

Statistical analyses were performed using the Statistical Package for the Social Sciences version 20.0 software (IBM Corp., NY, USA) [32]. The initial data were analyzed statistically using multifactorial analysis of variance (ANOVA) (two-tailed ANOVA) to determine the influence of seasonal variation, rodent species, and sex on parasitic infestation rates. We used the following statistical model:

Y_ijk_ = µ+α_i_+β_j_+(αβ)_ij_+ε_ijk_

Where Y_ijk_ is the dependent variable measurement; µ is the overall mean; α_i_ is the fixed effect of seasonal variation, β_j_ is the fixed effect of rodent type and sex, (αβ)_ij_ is the interaction of season and rodent species, and ε_ijk_ is the random error.

Pearson’s correlation was conducted to assess the relationships between macroclimatic conditions and rodent distribution and ectoparasitic infestation rates. Correlations were considered strong when r≥0.6, intermediate when 0.6<r≥0.4, and weak when r<0.4. The results were expressed as highly significant when p<0.01, significant when p≤0.05, and non-significant when p>0.05.

## Results

### Rodent identification and frequencies

In the current study, 380 rodents were captured during the four successive seasons. The numbers were concerning seasons that were; in summer about 186 (106 males and 80 females), fall about 71 (36 males and 35 females), winter about 39 (22 males and 17 females), and spring about 84 (50 males and 34 females). Four rodent species were identified and quantified: Rattus norvegicus (brown rat), n=161 (92 males and 69 females); *Rattus rattus frugivorus* (white-bellied rat), n=119 (67 males and 52 females); *Rattus rattus alexandrines* (gray-bellied rat), n=50 (27 males and 23 females); and Mus musculus *domesticus* (house mouse), n=50 (28 males and 22 females).

### Pp of ectoparasitic infestations

R. norvegicus revealed significantly (p<0.01) higher Pp rates as revealed in Table-1 in males during fall and spring and females during summer. *R. rattus frugivorus* revealed significantly (p<0.01, Table-1) higher Pp rates in males during fall and winter and females during summer. *R. rattus alexandrines* had significantly (p<0.01) higher Pp rates during fall and winter in males and winter in females (Table-1). M. musculus *domesticus* in Tabe-1 revealed significantly (p<0.01) higher Pp rates during winter and summer in males and winter, fall, and spring in females.

**Table-1:** Period prevalence of parasitic infestations in different captured rodent species with concern to rodent sex during different seasons.

Species	Sex	Measures	Seasons				p-value

			Summer	Fall	Winter	Spring	
*Rattus norvegicus*	M	Cap. No	47^a^	15^c^	7^d^	23^b^	0.000
		Infest. No	36^a^	14^c^	4^d^	21^b^	0.001
		Pp_infests_ %	76.6^b^	93.3^a^	57.1^c^	91.3^a^	0.000
	F	Cap. No	29^a^	15^b^	7^c^	18^b^	0.000
		Infest. No	27^a^	12^b^	3^c^	16^b^	0.000
		Pp_infests_ %	93.1^a^	80.0^c^	42.9^d^	88.9^b^	0.002
*Rattus rattus frugivorus*	M	Cap. No	37^a^	10^c^	4^d^	16^b^	0.001
		Infest. No	31^a^	10^c^	4^c^	15^b^	0.000
		Pp_infests_ %	83.8^c^	100.0^a^	100.0^a^	93.7^b^	0.002
	F	Cap. No	31^a^	7^c^	5^d^	9^b^	0.001
		Infest. No	27^a^	5^b^	3^b^	7^b^	0.001
		Pp_infests_ %	87.1^a^	71.4^b^	60.0^c^	77.8^b^	0.000
*Rattus rattus* *alexandrinus*	M	Cap. No	12^a^	4^b^	5^b^	6^b^	0.001
		Infest. No	10^a^	4^b^	5^b^	5^b^	0.002
		Pp_infests_ %	83.3^b^	100.0^a^	100.0^a^	83.3^b^	0.001
	F	Cap. No	8^b^	10^a^	2^c^	3^c^	0.002
		Infest. No	6^b^	8^a^	2^c^	2^c^	0.002
		Pp_infests_ %	75.0^c^	80.0^b^	100.0^a^	66.7^d^	0.000
*Mus musculus demesticus*	M	Cap. No	10^a^	7^b^	6^b^	5^b^	0.001
		Infest. No	8^a^	4^b^	5^b^	3^b^	0.000
		Pp_infests_ %	80.0^a^	57.1^c^	82.3^a^	60.0^b^	0.001
	F	Cap. No	12^a^	3^b^	3^b^	4^b^	0.001
		Infest. No	10^a^	3^b^	3^b^	4^b^	0.001
		Pp_infests_ %	83.3^b^	100.0^a^	100.0^a^	100.0^a^	0.002

Means carrying different superscripts in the same row are significantly different at (p≤0.05) or highly significantly different at (p<0.01). Means carrying the same superscripts in the same row are non-significantly different at (p>0.05). The total number of captured rodents=380 (186 in summer, 71 in fall, 39 in winter, and 84 in spring). The total number of infested rodents=324 (162 in summer, 60 in fall, 29 in winter, and 73 in spring). Cap. No=Number of captured rodents, Infest. No=Number of infested rodents, Pp _infestation_=Period prevalence of parasitic infestation, M=Male, F=Female

Flea Pp was significantly (p<0.01) higher (Table-2) during summer in males and fall in females R. norvegicus, fall in males and summer in females *R. rattus frugivorus*, and winter in males and females *R. rattus alexandrines* and M. musculus *domesticus*. Lice Pp (Table-2) was significantly (p<0.01) higher during winter in males and females R. norvegicus, *R. rattus frugivorus*, *R. rattus alexandrines*, and M. musculus *domesticus*. Mite Pp was significantly (p<0.01, Table-2) higher during spring in males and females R. norvegicus, *R. rattus frugivorus*, *R. rattus alexandrines*, and M. musculus *domesticus*.

**Table-2:** Period prevalence of various ectoparasitic infestations in captured rodent species in concern with sex during different seasons.

Species	Sex	Pp %	Seasons				p-value

			Summer	Fall	Winter	Spring	
*Rattus norvegicus*	M	Pp_Flea_	34.0^a^	29.2^a^	32.5^a^	19.7^b^	0.001
		Pp_Lice_	11.7^b^	15.6^b^	37.5^a^	3.3^c^	0.002
		Pp_Mite_	54.3^b^	55.2^b^	30.0^c^	77.0^a^	0.002
	F	Pp_Flea_	33.1^a^	31.6^a^	31.3^a^	15.4^b^	0.000
		Pp_Lice_	5.9^c^	12.3^b^	43.8^a^	6.4^c^	0.001
		Pp_Mite_	61.0^b^	56.1^c^	25.0^d^	78.2^a^	0.000
*Rattus rattus frugivorus*	M	Pp_Flea_	27.7^ab^	31.3^a^	24.0^bc^	20.9^c^	0.001
		Pp_Lice_	12.0^b^	16.7^b^	46.0^a^	5.2^c^	0.001
		Pp_Mite_	60.2^b^	52.0^c^	30.0^d^	73.9^a^	0.002
	F	Pp_Flea_	31.3^a^	30.6^a^	30.8^a^	13.9^b^	0.001
		Pp_Lice_	8.2^bc^	12.2^b^	38.5^a^	5.1^c^	0.000
		Pp_Mite_	60.4^b^	57.1^b^	30.8^c^	81.0^a^	0.001
*Rattus rattus alexandrinus*	M	Pp_Flea_	32.0^a^	24.1^b^	33.3^a^	20.0^b^	0.002
		Pp_Lice_	9.3^c^	16.1^b^	38.5^a^	5.9^c^	0.001
		Pp_Mite_	58.7	59.8	28.2	74.1	0.000
	F	Pp_Flea_	25.7^b^	17.7^c^	33.3^a^	12.5^d^	0.001
		Pp_Lice_	4.8^c^	14.5^b^	33.3^a^	3.8^c^	0.001
		Pp_Mite_	69.5	67.6	33.3	83.3	0.000
*Mus musculus demesticus*	M	Pp_Flea_	25.0^b^	25.9^b^	32.6^a^	22.1^b^	0.001
		Pp_Lice_	5.9^c^	13.8^b^	37.0^a^	7.1^c^	0.002
		Pp_Mite_	69.1^a^	60.3^b^	30.4^c^	70.7^a^	0.000
	F	Pp_Flea_	17.1^b^	21.4^ab^	25.0^a^	17.9^b^	0.001
		Pp_Lice_	3.8^b^	7.1^b^	43.2^a^	5.1^b^	0.000
		Pp_Mite_	79.0^a^	71.4^b^	31.3^c^	79.9^a^	0.001

Means carrying different superscripts in the same row are significantly different at (p≤0.05) or highly significantly different at (p<0.01). Means carrying the same superscripts in the same row are non-significantly different at (p>0.05). The total number of isolated flea=746 (345 in summer, 168 in fall, 70 in winter, and 163 in spring), the total number of isolated lice=328 (99 in summer, 90 in fall, 93 in winter, and 46 in spring), the total number of isolated mites=1856 (748 in summer, 372 in fall, 69 in winter, and 667 in spring). Pp_flea_=Period prevalence of flea infestation, Pp_lice_=Period prevalence of lice infestation, Pp_mite_=Period prevalence of mite infestation, M=Male, F=Female

### Ecological macroclimatic correlations

Temperature showed in Table-3 highly significant (p<0.01) positive correlations (r=0.562, 0.532, 0.495) with the total number of captured rodents, males, and females, respectively. Temperature also revealed highly significant (p<0.01) positive correlations (r=0.641, 0.511, 0.479) with the total number of infested rodents, males, and females, respectively.

**Table-3:** Pearson’s correlation between ecological macroclimatic conditions with the number of captured rodents in concern with sex (above diagonal) and with the number of infested rodents in concern with sex (below diagonal).

R	Temp.	RH	DP	Cap. rodents	Rodent Sex	

					Male	Female
Temp	1	−0.794**	0.676**	0.562**	0.532**	0.495**
RH	−0.794**	1	−0.420**	−0.666**	−0.680**	−0.528**
DP	0.676**	−0.420**	1	0.327**	0.253**	0.353**
Infes. Rodents	0.641**	−0.743**	0.320**	1	0.926**	0.903**
Rodent Sex						
Male	0.511**	−0.620**	0.224*	0.758**	1	0.674**
Female	0.479**	−0.596**	0.306**	0.609**	0.523**	1

** Correlations are highly significant at (p<0.01).

* Correlations are significant at (p≤0.05). ^NS^Correlations are non-significant at (p>0.05). Strong correlations at r≥0.6, Intermediate correlations at r≥0.4 and r<0.06, weak correlations at r<0.4. R=Person’s correlation coefficient, Temp=Temperature, RH=Relative humidity, DP=Dew point, Cap.=Total number of captured rodents (380 as 186 in summer, 71 in fall, 39 in winter, and 84 in spring), Infes.=Total No. of infested rodents (324 as 162 in summer, 60 in fall, 29 in winter, and 73 in spring)

Relative humidity showed highly significant (p<0.01, Table-3) negative correlations (r=−0.666, −0.680, −0.528) with the total number of captured rodents, males, and females, respectively. Relative humidity also showed highly significant (p<0.01) negative correlations (r=−0.743, −0.620, −0.596) with the total number of infested rodents, males, and females, respectively.

Dew point revealed in Table-3 highly significant (p<0.01) positive correlations (r=0.327, 0.253, 0.353, 0.320, 0.224, 0.306) with the captured rodents, males, females, infested rodents, infested males, and infested females numbers, respectively.

### Parasitological identification

Microscopic examinations revealed three main ectoparasites species: fleas (three identified species), lice (four identified species), and mites (four identified species).

**Figure-3:**
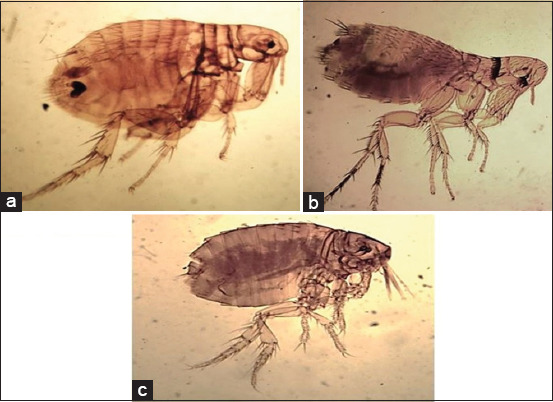
Light photomicrograph of a: *Xenopsylla cheopis*; b: *Leptopsylla segnis*; c: *Echidnophaga gallinacea* (×10).

The identified flea species were *Xenopsylla cheopis* (Figure-3a) that characterized by the absence of ctenidia, presence of a mesopleural rod, and presence of more than one post-occipital bristle. *Leptopsylla segnis* (Figure-3b) characterized by the presence of genal and pronotal ctenidia, and vertical genal ctenidia were composed of four elements. *Echidnophaga gallinacea* (Figure-3c) was characterized by the absence of ctenidia, a well-developed occipital lobe, presence of two post-occipital bristles, and a backward directed genal lobe.

**Figure-4:**
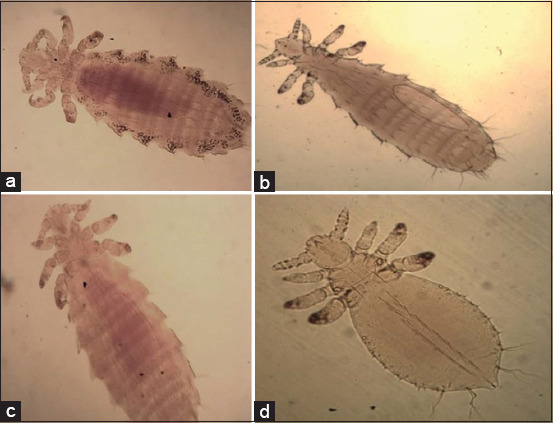
Light photomicrograph of a: *Hoplopleura ocanthopus* b: *Hoplopleura hirsuta*; c: *Hoplopleura oenomydis;* d: *Polyplax spinulosa* (×10).

The identified lice species were *Hoplopleura ocanthopus* (Figure-4a) that characterized by the presence of four to five large parategal plates with two large setae on the posterior margin. *Hoplopleura hirsuta* (Figure-4b) was characterized by the presence of six pairs of distal abdominal setae, seven tergal plates bearing a row of setae in males, and four to six slightly elongated paratergal plates in females. *Hoplopleura oenomydis* (Figure-4c) was identified by the presence of abdominal setae in the membrane between the sternal and paratergal plates. *Polyplax spinulosa* (Figure-4d) possess paratergal plates, among which three to five have a dorsal apical angle.

**Figure-5:**
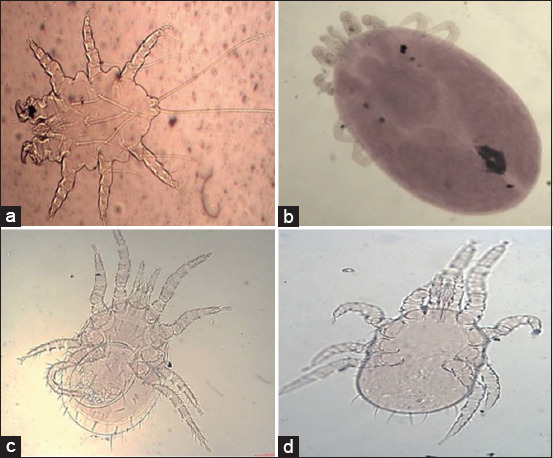
Light photomicrograph of a: *Myobia musculi*; b: *Dermanyssus gallinae*; c: *Laelaps nuttalli* d: *Ornithonyssus bacoti* (×40).

The mites were categorized into *Myobia musculi* (Figure-5a) which is characterized by the short first pair of legs and second pair of legs ending in empodia. *Dermanyssus gallinae* (Figure-5b) is characterized by a dorsal shield that does not reach the posterior margin and the presence of small setae on the dorsal shield and around the dorsal plate. *Laelaps nuttalli* (Figure-5c) is characterized by hypostomes with a dorsal labrum of two lobes covered with minute papillae, segmented chelicerae, and pulvillus terminated with two medioventral claws. *Ornithonyssus bacoti* (Figure-5d) has a dorsal plate that tapers gradually to blunt point setae of the same size and chelicera with no teeth.

### Species-specific Pp and RR

Flea Pp revealed in Table-4 significant (p<0.01) increases in *Echidnophaga gallinacean n*, *X. cheopis*, and *L. segnis* during summer with increased RR during winter. Lice Pp revealed in Table-5 significant (p <0.01) increases in the Pp of *H. hirsuta, H. ocanthopus, H. oenomydis*, and *P. spinulosa* during summer with high RR during winter. Mite Pp revealed also significant (p<0.01, Table-6) increases in the Pp of *L. nuttalli, D. gallinae, O. bacoti*, and *M. musculi* with high RR during summer.

**Table-4:** Period prevalence and relative risk of flea infestations in captured rodents during different seasons.

Flea species	Measures	Seasons				p-value

		Summer	Fall	Winter	Spring	
*Echidnophaga gallinacea*	No	109^a^	88^b^	38^d^	77^c^	0.002
	Pp	14.6^a^	11.8^b^	5.1^d^	10.3^c^	0.001
	RR	0.13^c^	0.18^b^	0.25^a^	0.11^c^	0.000
*Xenopsylla cheopis*	No	108^a^	67^b^	34^d^	44^c^	0.002
	Pp	14.5^a^	9.0^b^	4.6^c^	5.9^c^	0.000
	RR	0.12^b^	0.14^b^	0.22^a^	0.06^c^	0.000
*Leptopsylla segnis*	No	63^a^	53^b^	28^d^	37^c^	0.001
	Pp	8.4^a^	7.1^b^	3.8^d^	5.0^c^	0.000
	RR	0.08^bc^	0.11^b^	0.18^a^	0.05^c^	0.000

Means carrying different superscripts in the same row are significantly different at (p≤0.05) or highly significantly different at (p<0.01). Means carrying the same superscripts in the same row are non-significantly different at (p>0.05). The total number of captured rodents=380 (186 in summer, 71 in fall, 39 in winter, and 84 in spring). The total number of infested rodents=324 (162 in summer, 60 in fall, 29 in winter, and 73 in spring). The total number of isolated flea=746 (345 in summer, 168 in fall, 70 in winter, and 163 in spring). The number of isolated fleas species=E*chidnophaga gallinacea*; 312, *Xenopsylla cheopis*; 253, and *Leptopsylla segnis*; 181. No=Frequencies of the isolated parasites, Pp_=_Period prevalence, RR=Relative risk

**Table-5:** Period prevalence and relative risk of lice infestations in captured rodents during different seasons.

Lice species	Measures	Seasons				p-value

		Summer	Fall	Winter	Spring	
*Hoplopleura hirsuta*	No	57^a^	40^b^	25^c^	9^d^	0.000
	Pp	17.4^a^	12.2^b^	7.6^c^	2.7^d^	0.001
	RR	0.08^b^	0.08^b^	0.17^a^	0.01^c^	0.000
*Hoplopleura ocanthopus*	No	11^a^	9^b^	6^c^	4^c^	0.000
	Pp	3.4^a^	2.7^b^	1.8^c^	1.2^c^	0.000
	RR	0.01^b^	0.02^b^	0.05^a^	0.01^b^	0.001
*Hoplopleura oenomydis*	No	10^a^	7^b^	4^c^	3^c^	0.002
	Pp	3.0^a^	2.1^b^	1.2^c^	0.9^c^	0.000
	RR	0.01^a^	0.01^a^	0.03^a^	0.00^a^	0.001
*Polyplax spinulosa*	No	66^a^	39^b^	27^c^	11^d^	0.000
	Pp	20.1^a^	11.9^b^	8.2^c^	3.4^d^	0.001
	RR	0.01^c^	0.08^b^	0.19^a^	0.02^c^	0.001

Means carrying different superscripts in the same row are significantly different at (p ≤ 0.05) or highly significantly different at (p < 0.01). Means carrying the same superscripts in the same row are non-significantly different at (p > 0.05). The total number of captured rodents = 380 (186 in summer, 71 in fall, 39 in winter, and 84 in spring). The total number of infested rodents = 324 (162 in summer, 60 in fall, 29 in winter, and 73 in spring). The total number of isolated lice = 328 (99 in summer, 90 in fall, 93 in winter, and 46 in spring). The number of isolated lice species = *Hoplopleura hirsuta*; 131, *Hoplopleura ocanthopus*; 30, *Hoplopleura oenomydis*; 24, and *Polyplax spinulosa*; 143. No = Frequencies of the isolated parasites, Pp = Period prevalence, RR = Relative risk.

**Table-6:** Period prevalence and relative risk of mite infestations in captured rodents during different seasons.

Mite species	Measures	Seasons				p-value

		Summer	Fall	Winter	Spring	
*Laelaps nuttalli*	No	444^a^	277^b^	219^c^	166^d^	0.001
	Pp	23.9^a^	14.9^b^	11.8^c^	8.9^d^	0.000
	RR	0.55^a^	0.44^b^	0.32^c^	0.29^c^	0.000
*Dermanyssus gallinae*	No	163^a^	120^b^	86^c^	45^d^	0.001
	Pp	8.8^a^	6.5^b^	4.6^c^	2.4^d^	0.000
	RR	0.29^a^	0.23^b^	0.07^c^	0.10^c^	0.001
*Ornithonyssus bacoti*	No	104^a^	78^b^	57^c^	34^d^	0.002
	Pp	5.6^a^	4.2^b^	3.1^c^	1.8^d^	0.000
	RR	0.20^a^	0.16^b^	0.22^a^	0.08^c^	0.000
*Myobia musculi*	No	28^a^	20^b^	10^c^	5^d^	0.001
	Pp	1.5^a^	1.1^b^	0.5^c^	0.3^c^	0.000
	RR	0.06^a^	0.05^a^	0.03^b^	0.01^b^	0.000

Means carrying different superscripts in the same row are significantly different at (p≤0.05) or highly significantly different at (p<0.01). Means carrying the same superscripts in the same row are non-significantly different at (p>0.05). The total number of captured rodents=380 (186 in summer, 71 in fall, 39 in winter, 84 in spring). The total number of infested rodents=324 (162 in summer, 60 in fall, 29 in winter, and 73 in spring). The total number of isolated mites=1856 (748 in summer, 372 in fall, 69 in winter, and 667 in spring). The number of isolated mite species=L*aelaps nuttalli*; 1106, *Dermanyssus gallinae*; 414, *Ornithonyssus bacoti*; 273, and *Myobia musculi*; 63. No=Frequencies of the isolated parasites, Pp_=_Period prevalence, RR=Relative risk

## Discussion

Rodents are considered a worldwide public health threat as populations increase due to the availability of resources and suitable macroclimatic conditions, including temperature, relative humidity, and dew point in rural and urban areas [33]. Rodent’s populations can rapidly grow, resulting in extensive damage to electrical installations, properties, food stores, crops, and grains. The World Health Organization reported that 5-22% of the total global food production is lost by rodent activities [34]. Anthropogenic activities contribute to ecosystem modifications with subsequent changes in the ecological distribution and prevalence of rodents [35]. The parasitism interrelationship between rodents and ectoparasites is complicated. Higher prevalence of rodents might contribute to a higher risk of ectoparasitic infestation resulting in the transmission of more infectious and zoonotic diseases either directly from rodents or ectoparasites [36,37] or indirectly through exposure to urine, feces, saliva, and blood [38-40] or arthropod ectoparasites [41].

In the current study, we collected and identified four rodents: R. norvegicus (brown rat, n=161), *R. rattus frugivorus* (white-bellied rat, n=119), *R. rattus alexandrines* (gray-bellied rat, n=50), and M. musculus *domesticus* (house mouse, n=50). The seasonal prevalence revealed that R. norvegicus, *R. rattus frugivorus*, and M. musculus *domesticus* were predominant in the summer, and *R. rattus alexandrines* was predominant in both the summer and fall. The abundance of rodents in North Sinai is closely related to ecological and climatic conditions, food availability, and socioeconomics of the area. That is why the area suffers from extensive damage to the properties, rodent dropping could be seen everywhere, and some zoonotic diseases with low incidence were recorded. Our findings were consistent with Millán *et al*. [42] and Pollack *et al*. [43], who reported that ectoparasites are dependent on rodent survival which in turn usually require moderate temperatures and high relative humidity for survival, multiplication, and development. Yusefi *et al*. [44] reported that a synchronized pattern recorded a high burden of rodents in some terrestrial areas of Iran for the availability of resources and food. Soliman *et al*. [45] also noted that environmental conditions, such as the season, topography, and vegetation, as well the availability of food and water resources and hiding places affect rodent hosts and their ectoparasites.

Niche-fulfilling epidemiology has been used to explain the ecological distribution of ectoparasites based on host availability and distribution. Dziemian *et al*. [46] and Hamidi *et al*. [47] reported that the moderate atmospheric conditions and food availability that prevail in summer and spring encourage the growth and multiplication of rodents and their ectoparasites that might harbor highly zoonotic agents. Ectoparasitic infestations contribute to anemia and circulatory disorders, secondary infections, irritation, food wastage, lower production and reproduction, skin lesions, hide and wool deterioration that renders them non-marketable, and intoxication in small animals [48]. Moreover, an abundance of emerging diseases is transmitted through ectoparasites [49,50]. Gholipoury *et al*. [51] recorded the transmission of zoonotic and non-zoonotic parasitic diseases by rodents in Northeastern Iran. Eslami *et al*. [52] investigated the ectoparasitic infestation prevalence in *Rattus rattus* of Qeshm Island, Iran, attributing the high rates of infestation to ideal ecological conditions for host growth in the area.

The current study revealed that the Pp of fleas in North Sinai increased during summer in males and fall in females of R. norvegicus, fall in males and summer in females *R. rattus frugivorus*, and during winter in males and females *R. rattus alexandrines* and M. musculus *domesticus*. The identified fleas, *E. gallinacean*, *X. cheopis*, and *L. segnis*, prevailed during summer with an increased RR during winter. Our results were similar to Kowalski and Bogdziewicz [53], who reported that *X. cheopis*, which transmits *Y. pestis* and the human plague, contributes to endemic disease in some geographical areas as Southeast Asia. Shahraki *et al*. [54] also identified *X. cheopis* in Iran, and Hamidi and Nassirkhani [55] reported similar results and isolated and identified fleas from rodents in Iran. Dahesh *et al*. [56] reported the risk of rodent fleas in the transmission of *Trypanosoma* spp. Keskin *et al*. [57] recovered new host-associated fleas from rodents in Turkey.

The current results also revealed an increased Pp of lice during winter in R. norvegicus, *R. rattus frugivorus*, *R. rattus alexandrines*, and M. musculus *domesticus*. *H. hirsuta, H. ocanthopus, H. oenomydis*, and *P. spinulosa* prevailed during summer with an increased RR during winter. The results were consistent with Abdel-Rahman *et al*. [58], who reported a high prevalence of ectoparasitic burden (mainly lice) on M. musculus *domesticus* in Hail of Saudi Arabia.

Mite Pp increased during spring in R. norvegicus, *R. rattus frugivorus*, *R. rattus alexandrines*, and M. musculus *domesticus*. *L. nuttalli, D. gallinae, O. bacoti*, and *M. musculi* prevailed and showed an increased RR during summer. Mites are highly specialized and contagious ectoparasites that parasitize rodents. Shamsi *et al*. [59] recovered high rates of chigger mite (*Trombiculidae*) infestations in rodents in Iran. Similarly, Stekolnikov *et al*. [60] recorded four species of chigger mites recovered from rodents in Saudia Arabia. From another perspective, Eladl *et al*. [61] recorded a high prevalence of mites in laying hen farms in Egypt, reporting residues of pyrethroids in the egg contents.

Rodents can consume a variety of available foods in the ecosystem and tolerate changes well, allowing for rapid population growth [62]. These circumstances make rodents a perfect vector for harboring ectoparasites and transmitting infectious and zoonotic diseases. The extensive activities of humans increase the risk of direct contact with rodents in their habitat, contributing to the transmission of disease [63,64]. Combating rodent infestation should be considered to minimize the risk of transmitting disease through rodents and their infesting ectoparasites [65]. Combating measures can include mechanical control using traditional break-back traps or metal traps and physical barriers [66], biological control using natural enemies without altering the ecological balance [67,68], and chemical control using aluminum phosphide fumigant [69] or anticoagulants [70].

## Conclusion

The geographical location and predominating ecological conditions of North Sinai, Egypt, provide a suitable environment that encourages the growth of rodents, including R. norvegicus during summer, *R. rattus frugivorus* during fall, *R. rattus alexandrines* during winter, and M. musculus *domesticus* during spring. Fleas showed different predomination patterns according to season and rodent sex and species. Lice predominated during winter in male and female rodents. Mites predominated during spring in male and female rodents.

The identified ectoparasites were fleas (*E. gallinacean*, *X. cheopis*, and *L. segnis*) with increased risk during winter, lice (*H. hirsuta*, *H. ocanthopus*, *H. oenomydis*, and *P. spinulosa*) with increased risk during winter, and mites (*L. nuttalli*, *D. gallinae*, *O. bacoti*, and *M. musculi*) with increased risk during summer.

Strict preventive and biosecurity measures should be adopted in North Sinai, Egypt, to combat the increased number of rodents and high rates of ectoparasitic infestations. Such measures minimize the risk of transmitting some zoonotic diseases carried by rodents or infesting ectoparasites. Potential control measures might include mechanical, chemical, and biological controls. Overall, the present study established baseline data for rodent species and ectoparasitic fauna in North Sinai, which may facilitate appropriate planning on the control and prevention of rodents and zoonotic diseases in the region.

## Authors’ Contributions

DSF: Supervised the rodent collection from North Sinai and participated in writing of the manuscript. ESS: Designed the study, participated in samples collection, run the epidemiological measurements, and took part in writing of the manuscript. NHS: Conducted parasitological examinations and participated in writing of the manuscript. AMSE: Participated in the ectoparasites collection from rodents, rodents’ identification, and participated in writing of the manuscript. All authors read and approved the final manuscript.
